# mHealth and Perinatal Depression in Low-and Middle-Income Countries: A Scoping Review of the Literature

**DOI:** 10.3390/ijerph17207679

**Published:** 2020-10-21

**Authors:** Aliyah Dosani, Harshmeet Arora, Sahil Mazmudar

**Affiliations:** 1 School of Nursing and Midwifery, Faculty of Health, Community and Education, Mount Royal University, Calgary, AB T3E 6K6, Canada; 2 Department of Community Health Sciences, Cumming School of Medicine, University of Calgary, Calgary, AB T2N 1N4, Canada; 3 O’Brien Institute for Public Health, Cumming School of Medicine, University of Calgary, Calgary, AB T2N 1N4, Canada; 4 Department of Computer and Electrical Engineering, Faculty of Applied Science, University of British Columbia, Vancouver, BC V6T 1Z4, Canada; harshmeet.arora@alumni.ubc.ca (H.A.); sahil.mazmudar@alumni.ubc.ca (S.M.)

**Keywords:** mhealth, mobile phone health applications, perinatal depression, postpartum depression, antenatal depression, low- and middle-income countries, resource-poor countries

## Abstract

Women in low- and middle-income countries have high rates of perinatal depression. As smartphones become increasingly accessible around the world, there is an opportunity to explore innovative mHealth tools for the prevention, screening, and management of perinatal depression. We completed a scoping review of the literature pertaining to the use of mobile phone technologies for perinatal depression in low-and middle-income countries. PubMed CINHAL, and Google Scholar databases were searched, generating 423 results. 12 articles met our inclusion criteria. Two of the 12 articles reviewed mobile phone applications. The remaining 9 articles were study protocols or descriptive/intervention studies. Our results reveal that minimal literature is currently available on the use of mobile health for perinatal depression in low- and middle-income countries. We found four articles that present the results of an intervention that were delivered through mobile phones for the treatment of perinatal depressive symptoms and an additional qualitative study describing the perceptions of mothers receiving cognitive behavioral therapy via telephones. These studies demonstrated that depressive symptoms improved after the interventions. There is potential to improve the quality of mHealth interventions, specifically mobile phone applications for perinatal depressive symptoms and depression, through meaningful collaborative work between healthcare professionals and application developers.

## 1. Introduction

Perinatal depression occurs while a woman is pregnant or within 12 months of delivery [1]. While perinatal depression is common among diverse groups of women in many countries around the world [2,3,4,5], women in low- and middle-income countries experience a higher burden of this illness [6]. Recent studies have demonstrated that women in low- and middle-income countries have a perinatal depression rate of up to 48.5% [7,8,9,10,11]. These figures are alarming as the rates reported in high-income countries are much lower, ranging from 6.5% to 12.9% during the perinatal period [6,12]. Antenatal depression is a significant population health issue since it could result in potentially harmful impacts for the mother such as postpartum depression [13,14], and significant adverse outcomes for the infant including preterm birth [15,16,17,18] and delays in cognitive, language, and motor development [19] that could last for years to come [20]. Therefore, it is important to identify effective public health interventions that address perinatal depression in ways that are both accessible and acceptable to the population.

mHealth is a subset of eHealth that uses mobile technologies including advancements in innovative applications to address health priorities [21]. While the use of mHealth has been used to improve health services in a number of diseases for over a decade [22], its use in the area of perinatal depression is relatively new. Limited literature is currently available that describes how mHealth has been used with respect to perinatal depression. For example, in high-income countries, mHealth mobile phone applications have been used by women to self-report psychological well-being during pregnancy [23] and to screen for antenatal depression using either mobile phones [24,25,26] or an iPad [27]. In addition, mHealth has been tested to deliver various interventions including support for women at risk for depression post-discharge from hospital [26], including health information seeking on postpartum depression [28]. Mobile phones have been used to access self-help tools and emotional support for women with postpartum depression [29], as well as patient decision tools for making treatment choices [26]. mHealth has also been used to deliver counseling therapy via text messages [30], family therapy for young mothers with perinatal depressive symptoms [31], cognitive behavioral therapy for antenatal depression [32] and even to address the mental health concerns of fathers to be [33]. However, it is important to understand how the use of mHealth is developing in low- and middle-income countries where the potential for impact, in terms of improving perinatal outcomes, is high.

There are approximately 5 billion mobile phone subscriptions in the world [21]. Approximately 90% of the world’s population and 80% of people living in rural areas have access to mobile networks [34]. Adoption of smartphones in emerging markets (e.g., Brazil, India, Kenya, Nigeria) is driven largely by the younger population, where individuals between the ages of 18–34 comprise 60–80% of smartphone users [35]. In addition, in many low- and middle-income countries, mobile phone networks have evolved more rapidly than other infrastructure including paved roads and electricity [21]. Many people now have access to higher speeds of data transmission in conjunction with inexpensive and more powerful phones [21]. This improvement in available infrastructure presents an opportunity for mobile health applications to serve a demographic that is technologically able and agile that will form the majority of the population receiving healthcare in the decades to come. Furthermore, as our online interactions increase, such mobile health solutions have an opportunity to explore how individuals receive care remotely. Furthermore, many low-and middle-income countries are population-dense, with underdeveloped health systems, and are therefore more vulnerable to public health dangers such as the COVID-19 pandemic [36]. With the COVID-19 pandemic influencing how people interact in person, innovative mobile health solutions have an opportunity to transform how individuals receive care. The objective of our work was to conduct a scoping review of the literature to determine the availability of literature pertaining to the use of mHealth, specifically mobile phones, and perinatal depression in low- and middle-income countries.

## 2. Methods

**Figure 1:**
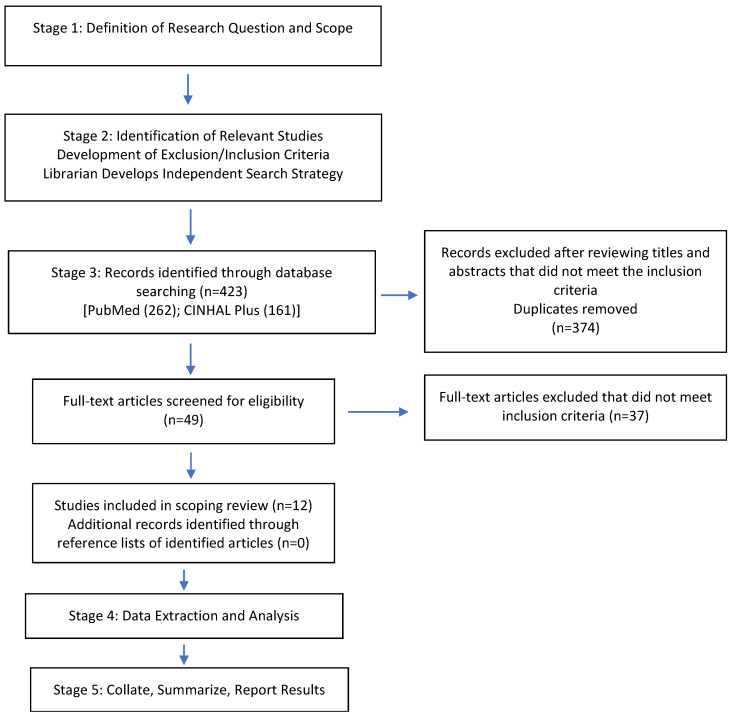
Stages of the scoping literature review.

We completed a scoping review of the literature to assess the status of available literature regarding mHealth and perinatal depression in low- and middle-income countries. Scoping reviews are exploratory and are used to examine the range and nature of a particular topic [37,38]. Specifically, this mapping exercise is used to determine the position of the literature and the magnitude of the research on a given topic [37]. We used Arksey and O’Malley’s [39] framework that outlines 5 stages for conducting a scoping review (Figure 1). Our inclusion criteria included articles that: (1) were published in peer-reviewed journals; (2) described the primary study population as women who were pregnant or had given birth in the past 12 months who reside in countries classified by the World Bank [40] as low- and middle-income countries; and/or pregnant women who were identified as users of mobile phone applications; (3) defined perinatal depression or anxiety as the outcome of interest; (4) discussed the use of mobile phones (including apps, text messaging-based interventions, or voice or video connection) for the prevention, screening, or treatment of perinatal depression. Articles were included irrespective of their research design and methodological quality, as prescribed by Valaitis and colleagues [38]. As such, we included study protocols, both quantitative and qualitative publications, and reviews of the literature. Exclusion criteria included non-English-language literature, commentaries, editorials, and theses. We searched PubMed, CINAHL Plus via EBSCOhost, and google scholar (Table 1) in July 2020. Our initial search of these databases generated 423 results. We excluded 374 articles after reviewing the titles and abstracts. From the remaining 49 articles, 37 were excluded as they did not meet the inclusion criteria. We included 12 articles for our analysis. Hand searching of the reference list of the remaining 12 articles produced no additional results. All of the 12 articles were published between 2015–2020.

## 3. Results

### 3.1. Articles Included

Of the 12 articles we included in our analysis, 3 (25%) were study protocols [41,42,43] and 2 (16.7%) were reviews of available mobile phone applications related to perinatal depression [44,45]. The 7 remaining articles (58.3%) were research studies [46,47,48,49,50,51,52]. These studies were heterogeneous in terms of how mobile phone technology was used (e.g., interactive text messaging, automated text messaging, automated voice mail or use of mobile phone application), the purpose of the use of mobile technology (prevention, screening, and/or treatment), the perinatal timeframe in terms of pregnancy or the postpartum period and population in terms of parity (Table 2, Table 3 and Table 4).

### 3.2. Population and Outcome Measure

In terms of the population of focus, 2 of the 12 articles (16.7%) were reviews of mobile phone applications that were intended to be used by women who were pregnant or in the postpartum period [44,45]. Five of the 12 articles (41.7%), 2 of which were study protocols [41,42,46,48,51], focused on pregnant women. One of these 5 articles (20%) included first-time expectant mothers only [46]. The other 5 articles (41.7%), including one study protocol, considered postpartum women [43,47,48,49,50,52]. Of these 5 articles, 2 (40%) included first time mothers [49,50]. Five of the 12 studies (41.7%) took place in China [43,44,46,48,49], followed by 3 studies (25%) in Iran [47,50,52]. The primary outcome measure of 8 of the 12 studies (66.7%) was the depressive symptoms of women who were pregnant or in the postpartum period [41,42,43,46,47,48,50,52]. Seven of these articles [42,43,46,47,48,50,52] described the use of the Edinburgh Postnatal Depression Scale to identify depressive symptoms and one article [41] described the use of the Patient Health Questionnaire—9 to quantify depressive symptoms. One additional study (8.3%) assessed the validity of the Edinburgh Postnatal Depression Scale when administered via telephone interviews [51].

### 3.3. Study Phase and Research Methods

Of the 12 articles, 3 (25%) were study protocols where data collection had not yet begun or was in progress [41,42,43]. Two of the 12 articles were reviews of mobile phone applications. One of these review articles evaluated the contents of all postpartum depression applications in China and their alignment with the guidelines for the prevention and treatment of postpartum depression [44] and the other evaluated mobile phone applications that were available on the Apple iTunes and Google Android play stores [45]. Of the remaining 10 articles, 9 (90%) were quantitative in nature [41,42,43,46,47,48,50,51,52] with 5 articles (55.6%) using randomized control trials as their research method [42,43,46,47,52]. Two of the 9 articles used cross-sectional study designs [48,51], one article utilized a single-case experimental design [41], and the other article used pre-test and post-test study design [50]. One article was qualitative [49] with another article including a qualitative component [41].

### 3.4. Findings

Four of the 12 articles (33.3%) described the completion of an intervention and the corresponding results [46,47,50,52]. Two of the 4 studies (50%) described mothers receiving support in the form of text messages [50] or verbal problem-solving support using mobile phones [52]. One of the four studies (20%) delivered cognitive-behavioral therapy modules as their intervention via mobile phone application [47]. The final article (20%) provided psychoeducation as the intervention using a mobile phone application [46]. In addition, mothers were able to interact with healthcare professionals using a platform within the application and ask questions related to pregnancy, childbirth, infant health, and care [46]. With respect to results, statistically, significant improvements in the Edinburgh Postnatal Depression scores were observed in all of the 4 studies after the implementation of the interventions described [46,47,50,52].

One of the 12 articles (8.3%) validated the use of the Edinburgh Postnatal Depression Scale using phone interviews [51]. The diagnosis of a major depressive episode was confirmed by diagnostic interviews with a Cronbach’s alpha coefficient of 0.861. An additional article (8.3%), found that 71.31% of their female participants used mobile phone applications for antenatal care with 46.11% identified as experiencing depressive symptoms [48]. Logistic regression analyses showed that depression was associated with the availability of disease-screening functions in the application and spending 30 min or more using the application [48]. The one qualitative article (8.3%) found that cognitive-behavioral therapy delivered via phone to help increase confidence in a maternal role, emotional control, and sense of support [49]. However, while the flexibility of delivery format was helpful given the busy schedules of new mothers, the authors found it difficult to meet the individual learning needs of new mothers [49]. One of the 2 review articles (50.0%) identified that postpartum depression mobile phone applications that are used in China do not include content that is known to be effective in terms of interventions or align with Chinese clinical practice guidelines in the management of postpartum depression [44]. Likewise, the other review article (50%) found that insufficient information is available regarding the authors or creators of the content in the postpartum depression mobile phone applications or where the information was obtained [45].

## 4. Discussion

This is the first study of its kind, that we are aware of, that reviewed the current state of the literature on mHealth and perinatal depression in low- and middle-income countries. The results of our study indicate that there is minimal literature currently available on the use of mobile health for perinatal depression in low- and middle-income countries. We found four articles that present the results of an intervention that were delivered through mobile phones for the treatment of perinatal depressive symptoms [46,47,50,52] and an additional qualitative study describing the perceptions of mothers receiving cognitive behavioral therapy via telephones [49]. These five studies have shown that both qualitative perceptions of depressive symptoms and quantitative scores of depressive symptoms improved after interventions using mobile phones. While these early studies are promising, more research needs to be conducted with respect to women living in resource-poor settings, particularly in more rural areas, who typically do not have access to specialty perinatal health services that are more easily accessible in urban centers [53]. This work is especially important given that at least half of the world’s population cannot access basic health services [54]. The increased use of mobile phones in rural regions of the world [34] can provide an opportunity to leverage technology to improve outcomes for women with depressive symptoms in the perinatal period.

de Figueiredo and colleagues [51] found that screening for perinatal depression using the Edinburgh Postnatal Depression Scale via the telephone to be both reliable and valid in the Brazilian context. Similar work is necessary for other low- and middle-income countries. Other authors have found ways to screen for perinatal depression using mobile phones [24,25,26]. However, what remains unclear is the reliability and validity of using the Edinburgh Postnatal Depression Scale when self-administered by women through a mobile phone application. Comparable analyses are required for other scales used to screen for perinatal depressive symptoms such as the Patient Health Questionnaire-9. Furthermore, the acceptability of telephone screening versus self-administered questionnaires via mobile phone applications needs to be determined to move research in the area of mHealth and perinatal depression forward in a meaningful way.

mHealth has the potential to become an important service link between the inadequate mental health services available in many parts of the world, particularly resource-poor settings, and the unfulfilled mental healthcare needs of a large number of people worldwide [55]. Our review shows that there are a myriad of mobile phone applications related to perinatal depression that are currently available for women to use in low- and middle-income countries [44,45] and that women in some countries, such as China, are beginning to increasingly use mobile phone applications for perinatal care more generally [48]. However, mobile phone applications currently available lack consistency with respect to the type and quality of information available [44,45]. Improvements in the innovative use of mHealth are critical to addressing perinatal depression in women in many parts of the world. Zhang and colleagues [45] found that current mobile phone applications lacked transparency in terms of both authorship and the evidence-base that framed the components of mobile phone applications, even some lacking references, and information where expected. This lack of transparency indicates a very real and potentially dangerous practice gap between healthcare professionals and application developers [44]. Such disjointed efforts between the knowledge holders, knowledge brokers, and mobile phone application developers ultimately results in unidimensional applications that fail to translate the expertise of healthcare professionals into user-friendly and intuitive mobile applications, and more importantly, those that result in desired outcomes. As such, increased knowledge-sharing efforts are required to develop applications that are not only evidence-based but effective in the prevention, screening, and management of perinatal depression.

With access to smart devices growing exponentially, there is a need for open-source projects where the software for the original source code for the mobile phone applications are made freely available and may be redistributed and modified according to the requirement of the user [56]. Open-source projects not only promote a platform for inclusive discussion amongst social innovators, but also allow for pooling resources, and partnerships. Using this open-source approach could also help to avoid barriers to entry due to licensing requirements and intellectual property regulations. Collaboration with open-source coding also has the potential to reduce the cost of designing and implementing the systems, while sharing data and information to improve efficiency and efficacy [56]. In addition, instead of a multitude of potentially unhelpful mobile phone applications being widely available, collaborators in this field could ensure that the applications are evidence-based. Furthermore, while most women who are pregnant or postpartum rely on user evaluations to select a mobile phone application [48], advocating for open-source software could not only improve the quality of applications but ensure that those delivering care can recommend evidence-based mobile phone applications to their clients and patients. Open source eHealth tools have been begun to gain traction in some resource-poor countries [57]. For example, Papadimitriou and colleagues [58] have demonstrated the success of using open source software to screen for mental health concerns in Kashmir. Making perinatal mental health mHealth tools open source would have the potential to increase positive outcomes exponentially.

A limitation of our scoping review of the literature is we chose to exclude articles about the perspectives of those delivering care using mhealth tools. While taking the perspectives of those delivering care, both trained medical professionals and skilled community health workers, is a significant component of understanding the successes and challenges of using mhealth, this was outside of the scope of our work. Studying the perspectives of health care providers or skilled community health workers who are tasked with delivering care is important in moving mhealth initiatives forward and, while related, is nuanced enough to be a separate field of study.

## 5. Conclusions

Minimal literature is currently available on the use of mobile health for perinatal depression in low- and middle-income countries. Research is currently in its early stages with respect to understanding the outcomes of interventions related to perinatal depression delivered via mobile phones. There is an immense amount of opportunity available to build meaningful relationships for health care professionals and mobile phone application developers. Such partnerships can ensure that the work undertaken is transparent and evidence-based to maximize the potential benefits of mHealth.

## Figures and Tables

**Table 1:** Search strategy.

Category	Search Parameters
Population	(“Pregnancy”[Mesh] OR “pregnancy”[tw] OR “pregnancies”[tw] OR “pregnant”[tw] OR “Postpartum period”[Mesh] OR “postpartum”[tw] OR “post-partum”[tw] OR “post partum”[tw] OR “puerperium”[Mesh] OR “puerperium”[tw] OR “Perinatal care”[Mesh] OR “perinat*”[tw] OR “postnat*”[tw] OR “maternal”[tw] OR “Prenatal Care”[Mesh] OR “prenatal”[tw] OR “Prenatal education”[Mesh] OR “antenatal” OR “Puerperal Disorders”[Mesh]) AND
Intervention	(“mobile health”[tw] OR “mhealth”[tw] OR “ehealth”[tw] OR “m-health”[tw] OR “e-health”[tw] OR “mcare”[tw] OR “Cell Phones”[Mesh] OR “Computers, Handheld”[Mesh] OR “cell phones”[tw] OR “cell phone”[TW] OR “cellular phone”[tw] OR “cellular phones”[tw] OR “cellular telephone”[tw] OR “cellular telephones”[tw] OR “mobile phone”[tw] OR “mobile phones”[tw] OR “mobile telephone”[tw] OR “mobile telephones”[tw] OR “iphone”[tw] OR “ipad”[tw] OR “cellphone”[tw] OR “cellphones”[tw] OR “pda”[tw] OR “personal digital assistant”[tw] OR “blackberry”[tw] OR “android”[tw] OR “smartphone”[tw] OR “smartphones”[tw] OR “smart phone”[tw] OR “smart phones”[tw] OR “tablet”[tw] OR “handheld computer”[tw] OR “apps”[tw] OR “mobile application”[tw] OR “mobile applications”[tw] OR “mobile communication”[tw] OR “mobile technology”[tw] OR “mobile games”[tw]) AND
Outcome	(“depression”[Mesh] OR “depression”[TW] OR “depressed”[TW] OR “depression, postpartum”[Mesh] OR “mental health”[Mesh] OR “mental health”[TW] OR “Mental disorders”[Mesh] OR “mental disorder”[TW] OR anxiety[TW] OR “Anxiety Disorders”[Mesh] OR “Anxiety”[Mesh])

**Table 2:** Data extraction: study design, study population, and details of the intervention.

First Author, Year, Country	Study Design	Number of Participants (*n*-)	Study Population/Data Collection	Identification of Depressive Symptoms and Cut-Off Scores	Depression Assessment Time Points
Chan et al., 2019 [46], China	Single-blind randomized control trial	*n* = 660 pregnant women (*n*-intervention = 330 and *n*-control = 330)	All first-time expectant mothers. Less than 24 weeks of gestation remaining. Attending the antenatal clinic at a public hospital	Validated Chinese version of the Edinburgh Postnatal Depression Scale (EPDS). No cut-off scores were provided.	First visit to antenatal clinic and follow-up at 4 weeks postpartum
Green et al., 2019 [41], Kenya	Single-case experimental design and qualitative interviews	Sample size has not been reported.	At least 20 weeks’ gestation or no more than 6 months postpartum. Recruited pregnant women and new mothers from 2 large public hospitals in Kiambu County, Kenya that offer SMS programs that promote healthy motherhood.	Patient Health Questionnaire-9 (PHQ-9), and a question about mood on a 10-point scale.	Participants are randomized to a 1- or 2-week baseline period and then invited to begin using Zuri. Participants are prompted to rate their mood via SMS every 3 days during the baseline and intervention periods.
Gureje et al., 2015 [42], Nigeria	Randomized control trial	*n* = 686 pregnant women	Pregnant women between the ages of 16 and 45 years. Gestational age between 16 and 28 weeks. Conducted in 29 clinics in Oyo State, Nigeria	-EPDS score ≥ 12 -Confirmed DSM-IV diagnosis of depression using relevant questions from the Composite International Diagnostic Interview	Assessments will be undertaken at baseline, 2 months following recruitment into the study, and 3, 6, 9, and 12 months after childbirth.
Jannati et al., 2020 [47], Iran	Non-blinded parallel-group randomized controlled trial	*n* = 78 new mothers (*n*-intervention = 39 and *n*-control = 39)	Women aged 18 or above. Given birth within the last 6 months. Attended one of three health care lefts in Kerman, Iran	EPDS score ≥13 Confirmed diagnosis of postpartum depression within two weeks after the participants’ recruitment using the International Neuropsychiatric Interview (MINI)	Assessments were undertaken at baseline and 2 months after baseline.
Li et al., 2020 [44], China	Analyzed and evaluated the contents of all postpartum depression applications (iOS and Android) in China	*n* = 19 applications iPhone (*n* = 6) Android (*n* = 2) WeChat (*n* = 11)	2 commentators used the PPD -related keywords to search for three application platforms in the Chinese market: Android, iOS, and WeChat, simplified Chinese and English.	Not applicable	Not applicable
Mo et al., 2018 [48], China	Cross-sectional study	*n* = 1304 pregnant women	Pregnant women who attended Hunan Provincial Maternal and Child Health Hospital.	EPDS score ≥10	One questionnaire was completed upon recruitment into the study
Ngai et al., 2019 [49], China	Qualitative study with semi-structured interviews analyzed by content analysis.	*n* = 39 first time mothers	A sample of 39 women from 197 was invited for semi-structured telephone interviews at 6 weeks postpartum.	EPDS score ≥10	Not applicable
Niksalehi et al., 2018 [50], Iran	Single-group, pre-test, and post-test study design.	*n* = 56 postpartum mothers	-Healthy first-time mothers recruited from a medical university-affiliated hospital in Bandar Abbas city, Iran. -Healthy and live singleton neonates born at 37–41 weeks of gestation.	EPDS score ≥12	Data were collected at baseline (14 days after giving birth) one week after receiving the intervention for 35 days.
de Figueiredo et al., 2015 [51], Brazil	Cross-sectional study	*n* = 496 pregnant women (*n*-cases = 257 and *n*-control = 239)	Pregnant women from 25 to 28 weeks of gestational age. Enrolled in prenatal care outpatient. services in the city of Ribeirão Preto, Brazil. 257 of 1083 participants (23.7%), had an EPDS score of ≥10. All participants were invited to visit the clinical research unit for a diagnostic interview with a psychiatrist or psychologist. After matching by age and date of delivery, 239 women with EPDS scores lower than 10 were also invited to a face-to-face interview.	Edinburgh Postnatal Depression Scale (EPDS score ≥10)	During the first year after childbirth, the women enrolled in the original study were contacted by telephone and invited to answer the EPDS. At the time of the diagnostic interview (SCID), the mothers completed the EPDS again, but in a self-administered format.
Shamshiri Milani et al., 2015 [52], Iran	Randomized control trial	*n* = 54 postpartum women (*n*-intervention = 27 and *n*-control = 27)	Postpartum women who had term deliveries, live births were uncomplicated deliveries. 54 eligible mothers out of 203 postpartum mothers (*n* = 27 per group) who had mild to moderate depression (>10 to˂14 EPDS Scores). These cases were recruited and randomly assigned to the intervention and control groups.	Edinburgh Postnatal Depression Scale (EPDS score ≥ 10)	10–15 days after childbirth and 6 weeks postpartum
Sun et al., 2019 [43], China	Study protocol for a double-blind randomized controlled trial	*n* = 120 postpartum women (*n*-intervention = 60 and *n*-control = 60)	Postpartum women up to 6 weeks post-delivery with EPDS score ≥9–≤12, selected randomly from one health left in each district within Changsha city.	Edinburgh Postnatal Depression Scale (EPDS score ≥9)	Baseline (t_0_), immediately after the last intervention (t_1_), 3 months following the intervention (t_2_), and 6 months following the intervention (t_3_).
Zhang et al., 2017 [45], Singapore	Evaluation of mobile phone applications to determine the quality of information presented for postnatal depression.	*n* = 11 android mobile phone applications *n* = 3 apple mobile phone applications	Apple iTunes and Google Android Play store applications searched. Inclusion criteria for applications: “postnatal”, “pregnancy”, “perinatal”, “depression”, “postpartum”, and must be in English.	Not applicable	Not applicable

**Table 3:** Data extraction: outcomes.

First Author, Year	Intervention and Comparator Group	Primary Outcome Measure	Other Outcome Measures
Chan et al., 2019 [46]	A mobile phone application called iParent, in addition to in-person nurse-led antenatal classes. Expectant mothers were able to ask questions that were answered by obstetricians via private, direct messages within the application and then shared in the Frequently Asked Questions module of the application.	The difference in the levels of antenatal and postnatal depression	Differences between anxiety levels, stress levels, and health-related quality of life before and after the RCT. Anxiety and stress levels were assessed with the Anxiety and Stress subscales of the Depression Anxiety Stress Scale (DASS). Health-related QoL was measured by the 12-item Short-Form Health Survey (SF-12).
Green et al., 2019 [41]	Automated the Thinking Healthy program via a mobile phone app called Healthy Moms over 15 sessions. During each Healthy Moms session, women will interact with the automated system via SMS.	Depression severity and mood	Participant engagement with the mobile phone application, intervention feasibility, and acceptability, variability in patient response to treatment.
Gureje et al., 2015 [42]	Intervention uses the WHO Mental Health Gap Action Programme Intervention Guide (mhGAP-IG) as adapted for the health system of Nigeria. Eight weekly sessions were delivered with case-specific additional sessions following childbirth. General physicians and psychiatrists provided mobile phone supervision, along with automated notifications to remind mothers of appointments and tasks.	The primary outcome is recovery from depression (EPDS < 6) at 6 months	Disability as measured by the WHO and the Disability Assessment Scale. Parenting skills using the Maternal Adjustment and Maternal Attitudes Questionnaire (MAMAs). Infant Toddler version of the Home Inventory for Measurement of the Environment. (HOME-IT) Maternal attitudes, the experience of stigma by mothers with the 12-item Discrimination and Stigma Scale. Health care utilization using The Client Service Receipt Inventory-PND version. Infant physical and cognitive development assessed using Bayley’s Scales.
Jannati et al., 2020 [47]	Mobile phone-based cognitive-behavioral therapy (CBT) on postpartum depression called Happy Mom, with eight weekly lessons. Lessons are structured as a storybook for mothers to follow and learn lessons from. Participants were randomized 1:1 to either the intervention group (mobile application access) or the control group (no mobile application access).	EPDS score post-intervention	None
Li et al., 2020 [44]	Currently available Chinese mobile phone applications for postpartum depression.	The adherence of mobile phone applications with clinical practice-based guidelines.	The Mobile App Rating Scale (MARS) to evaluate engagement, functionality, aesthetics of the application features.
Mo et al., 2018 [48]	No intervention as this was a descriptive study.	Use of antenatal care mobile phone applications and antenatal depression.	None
Ngai et al., 2019 [49]	No interventions as this was a qualitative study.	Specific components of the T-CBT intervention that women considered helpful in their preparation for early motherhood.	Not applicable
Niksalehi et al., 2018 [50]	Mobile phone SMS support for mothers with postpartum depression. Each mother received two daily text messages (morning and evening) for 35 days. Mothers could respond with a message or call the health care providers in the research team (a nurse and a midwife).	Depressive symptoms measured by the EDPS.	The satisfaction level of participants with the support received.
de Figueiredo et al., 2015 [51]	EPDS administered by telephone interviews. Each potential case (EPDS ≥ 10) was invited to a face-to-face diagnostic interview. The rest of the participants (EPDS < 10) were selected as controls and matched with potential cases by age and date of delivery.	The reliability and validity of the EPDS were administered by telephone interviews.	None
Shamshiri Milani et al., 2015 [52]	The intervention group received telephone support provided by eight healthy volunteers who were trained to communicate effectively with mothers to manage their problems. Each volunteer telephoned 3 to 4 mothers at intervals of 2 to 3 times per week until 6 weeks after childbirth.	EPDS score post-intervention	None
Sun et al., 2019 [43]	Six CBT modules were delivered via mobile phone application to participants over six weeks. Each module includes learning content and assignments. Participants in the control group will also complete six health education modules using the mobile phone application which follows the standard of care in the postpartum period and the child health management.	Postpartum depression	Negative emotion symptoms measured by the depression, anxiety, and Stress Scale (DASS-21) Parenting confidence as measured by the Chinese version of the Parenting Sense of Competence Scale (C-PSOC).
Zhang et al., 2017 [45]	Silberg Scale was used in the assessment of the information quality of smartphone applications.	Information quality score of mobile applications	None

**Table 4:** Data extraction: results and limitations.

First Author, Year	Attrition and Adherence	Results (Key Findings)	Limitations
Chan et al., 2019 [46]	At the follow-up T2 survey after the intervention, the retention rates were 66.1% (n = 218) for the intervention group and 68.2% (n = 225) for the control group.	Associations found between: 1. participation in the intervention and reduced depression 2. attendance in TAU classes and increased stress levels	The short postpartum period after which the follow-up assessment was conducted and the inclusion of first-time mothers rather than all mothers.
Green et al., 2019 [41]	Not applicable	Study protocol—no results were reported.	Recruited women who live in urban and peri-urban lefts in one part of Kenya, thus forgoing generalization of the broader population of Kenyan women.
Gureje et al., 2015 [42]	Not applicable	Study protocol - no results were reported.	None reported
Jannati et al., 2020 [47]	No information provided	Before the intervention, there was no statistically significant difference between the EPDS score between the two groups (*p* > 0.001). The average EPDS score after intervention was 8.18 and in the control group was statistically significant at 15.05.	The small sample size necessitates replication. Some women could have forgotten to study the sessions provided in the mobile application. This limitation was addressed by sending SMS reminders every week. This research did not obtain information in the intervention group on the setting, concentration level, and distractibility. Evaluation was carried out over two months, and the long-term effects of this application on the mood of the mothers need to be investigated.
Li et al., 2020 [44]	Not applicable	Postpartum depression applications in China lack known effective intervention content. Study suggests that to produce quality mobile apps, mental health professionals should be involved when adopting evidence-based guidelines for the prevention of postpartum depression.	There are no recent guidelines for the prevention of postpartum depression in China (latest in 2014) Only determines the existence or absence of clinical guidelines, rather than the extent of their effectiveness. Most applications lacked quality user feedback.
Mo et al., 2018 [48]	Not applicable	71.31% (930/1304) of the pregnant women used mobile phone applications for antenatal care. Higher usage of such applications was associated with urban residency, non-migrant status, first pregnancy, planned pregnancy, having no previous children, and wanting to communicate with peer pregnant women. 46.11% (601/1304) of pregnant women had depression. Logistic regression analyses showed that depression was associated with the availability of disease-screening functions in the apps (OR 1.78, 95% CI 1.03–3.06) and spending 30 min or more using the app (OR 2.05, 95% CI 1.19–3.52).	A cross-sectional study design led to limited data extrapolation, lacking causal inference. The demographic questionnaire used in this study was not tested for reliability and validity. The authors found heterogeneity in the types of antenatal care apps used by pregnant women in their sample.
Ngai et al., 2019 [49]	None	Majority of mothers found T-CBT helpful in increasing confidence in their maternal role, increased emotional control, and an increased sense of support. Facilitators of T-CBT included delivery of the therapy by a health care professional and the accessibility and flexibility of T-CBT. Busy schedule of new mothers and difficulty in meeting individual learning needs hindered the effectiveness of T-CBT. Most mothers would like the T-CBT to be extended over a longer period of time.	The results of this study may not be generalizable due to the small sample size.
Niksalehi et al., 2018 [50]	56 women were initially enrolled and n = 2 were lost to follow-up. N = 54 women were included in the analyses.	Mean score of EDPS pre-intervention was 14.44 (SD = 2.66). Mean post-intervention score was 11.94 (SD = 2.49). Mean overall decrease in scores on the EPDS pre- and post-intervention items was 2.5 points (*p* < 0.001). Majority of women (n = 26 [48.1%]) were moderately satisfied with text massages treatment delivery, followed by low satisfaction (n = 21 [38.9%]), and high satisfaction (n = 7 [13%]).	The single-group and pre–post-study design that may have resulted in Selection bias resulting in a homogenous sample that limits the generalizability of the results. Researchers used the self-administered EPDS tool for a postpartum depression diagnosis.
de Figueiredo et al., 2015 [51]	161 mothers who had EPDS ≥ 10 withdrew from the study 161 mothers who had EPDS ˂ 10 withdrew from the study. Therefore, n = 199 pregnant women (n-cases = 96 and n-control = 103) were included in the analyses.	In 90 participants, the diagnosis of the major depressive episode was confirmed by the diagnostic interview (EPDS ≥10 = 65; EPDS ˂10 = 23). The Cronbach’s alpha coefficient was 0.861. The Spearman’s correlation between the EPDS administered by telephone and the self-reported EPDS was 0.69 (*p* ˂ 0.001). The receiver-operating characteristic (ROC) curve for the EPDS administered by telephone was 0.78 (95% confidence interval (CI) = 0.72 to 0.84). Scores ≥ 10 showed a sensitivity of 72.2%, a specificity of 71.6%, and a positive predictive value of 67.7%. The application of a telephone interview represents a method to reduce the underdiagnosis, undertreatment, and harmful impact of postnatal depression for women, children, and families.	The large number of subjects who did not attend the diagnostic assessment (61.3%) despite multiple attempts to schedule the face-to-face interviews.
Shamshiri Milani et al., 2015 [52]	There were 5 participants from the intervention group and 3 from the control group that were lost to follow up. Therefore n = 46 women (n-intervention = 22 and n-control = 24) were included in the analyses.	Mean depression scores before intervention in both groups were the same. After intervention, the mean depression scores were 7.95 ± 3.45 for the intervention group and 10.33 ± 3.93 for the control group, which was statistically significant (*p* = 0.035). Changes in mean depression scores for both the intervention (−4.73 ± 3.83, *p* ≤ 0.001) and control (−2.5 ± 3.51, *p* = 0.008) groups were statistically significant. After the intervention, the mean depression scores in the intervention group who received telephone support was significantly lower than the control group.	The study did not include support from family and husband as an important factor in postpartum depression.
Sun et al., 2019 [43]	Not applicable	Study protocol—no results reported.	None reported
Zhang et al., 2017 [45]	Not applicable	14 applications are specifically focused on postnatal depression and were reviewed. The average score for the Silberg Scale of these applications was 3.0/9.0. Limited information is available about the creators or authors of the application, and references for the information included in the application itself. There is a need for healthcare professionals and developers to jointly conceptualize new applications with better information quality.	Authors identified applications from Apple or the Android application stores, potentially missing out on other sources. The search strategy was limited to applications in English and did not evaluate the multiple applications that are available in other languages. The Silberg Scale has not been validated for the assessment of information quality for smartphone applications. The Silberg Scale does not consider other aspects that may be relevant for smartphone application reviews, such as usability and levels of engagement.
